# Lysosome-related genes predict acute myeloid leukemia prognosis and response to immunotherapy

**DOI:** 10.3389/fimmu.2024.1384633

**Published:** 2024-05-10

**Authors:** Peng Wan, Liang Zhong, Lihua Yu, Chenlan Shen, Xin Shao, Shuyu Chen, Ziwei Zhou, Meng Wang, Hongyan Zhang, Beizhong Liu

**Affiliations:** ^1^ Central Laboratory of Yongchuan Hospital, Chongqing Medical University, Chongqing, China; ^2^ Key Laboratory of Laboratory Medical Diagnostics, Ministry of Education, Department of Laboratory Medicine, Chongqing Medical University, Chongqing, China; ^3^ Clinical Laboratory of Yongchuan Hospital, Chongqing Medical University, Chongqing, China

**Keywords:** acute myeloid leukemia, lysosome, prognostic model, immune infiltration, chemotherapy

## Abstract

**Background:**

Acute myeloid leukemia (AML) is a highly aggressive and pathogenic hematologic malignancy with consistently high mortality. Lysosomes are organelles involved in cell growth and metabolism that fuse to form specialized Auer rods in AML, and their role in AML has not been elucidated. This study aimed to identify AML subtypes centered on lysosome-related genes and to construct a prognostic model to guide individualized treatment of AML.

**Methods:**

Gene expression data and clinical data from AML patients were downloaded from two high-throughput sequencing platforms. The 191 lysosomal signature genes were obtained from the database MsigDB. Lysosomal clusters were identified by unsupervised consensus clustering. The differences in molecular expression, biological processes, and the immune microenvironment among lysosomal clusters were subsequently analyzed. Based on the molecular expression differences between lysosomal clusters, lysosomal-related genes affecting AML prognosis were screened by univariate cox regression and multivariate cox regression analyses. Algorithms for LASSO regression analyses were employed to construct prognostic models. The risk factor distribution, KM survival curve, was applied to evaluate the survival distribution of the model. Time-dependent ROC curves, nomograms and calibration curves were used to evaluate the predictive performance of the prognostic models. TIDE scores and drug sensitivity analyses were used to explore the implication of the model for AML treatment.

**Results:**

Our study identified two lysosomal clusters, cluster1 has longer survival time and stronger immune infiltration compared to cluster2. The differences in biological processes between the two lysosomal clusters are mainly manifested in the lysosomes, vesicles, immune cell function, and apoptosis. The prognostic model consisting of six prognosis-related genes was constructed. The prognostic model showed good predictive performance in all three data sets. Patients in the low-risk group survived significantly longer than those in the high-risk group and had higher immune infiltration and stronger response to immunotherapy. Patients in the high-risk group showed greater sensitivity to cytarabine, imatinib, and bortezomib, but lower sensitivity to ATRA compared to low -risk patients.

**Conclusion:**

Our prognostic model based on lysosome-related genes can effectively predict the prognosis of AML patients and provide reference evidence for individualized immunotherapy and pharmacological chemotherapy for AML.

## 1 Introduction

Acute myeloid leukemia (AML) is a highly invasive and destructive hematological malignancy and characterized by abnormal proliferation of hematopoietic cells and early blockage of myeloid differentiation, which impairs normal hematopoiesis with fatal consequences (1). For the past 40 years, the treatment regimen for AML has remained the standard induction chemotherapy regimen based on anthracyclines. Although the majority of patients experience complete remission after initial treatment, the presence of relapses and refractory events results in a 5-year survival rate below 30% (2). Advances in sequencing technology have helped us to gain insights into the pathogenesis of AML and accordingly develop new drug targets and formulate risk stratification, such as Fms-like tyrosine kinase 3 - internal tandem duplication (FLT3-ITD), Isocitrate dehydrogenase(IDH) mutations (3–5). The crosstalk between multiple genetic variants and the lack of clarity on the specific mechanisms of AML development ultimately leads to a mismatch between risk stratification and clinical outcomes, which in turn affects the quality of survival of AML patients (6). Therefore, it is urgent and necessary to further study the pathogenesis of AML, develop appropriate risk assessment methods and improve risk stratification.

Lysosomes are organelles produced by the Golgi apparatus that contain a variety of hydrolytic enzymes and have a unique ph value (7). Previous studies generally regarded lysosomes as organelles that break down substances, but in recent years, studies have pointed out that they not only break down substances and replenish nutrient metabolism, but also influence cell growth, disease generation, tumor progression, and other biological processes by mediating cellular signaling and participating in autophagy (8, 9). During tumor progression, lysosomal function undergoes a significant up-regulation to meet the energy demands necessary for the excessive proliferation and invasion of cancer cells (10). In contrast to normal cells, cancer cells exhibit a greater abundance and larger size of lysosomes, along with elevated lysosomal enzyme activities. Several lysosomal enzymes, such as cathepsin B and cathepsin D, besides their known role in mediating programmed cell death, are strongly implicated in poor patient prognosis (11–15). Additionally, the lysosomal fusion derivative known as Auer rods is predominantly observed in hematologic tumors, with current research focusing on their utility as diagnostic markers (16). However, the functional significance of this lysosomal derivative in acute myeloid leukemia remains poorly understood. Based on the aforementioned evidence, we hypothesize that the expression levels of lysosome-related genes could be utilized to categorize AML patients into distinct molecular subtypes, thereby guiding AML risk stratification and prognosis.

In our research, we collected lysosomal genes, constructed a prognostic model based on lysosome-related genes through systematic analysis, and conducted a preliminary validation of the model’s accuracy and usefulness. The aim is to improve the prognosis of AML and provide new reference evidence for individualized treatment of AML.

## 2 Methods

### 2.1 Data download and pre-processing

All data used in this study were obtained from two high-throughput sequencing platforms, TCGA(https://portal.gdc.cancer.gov/) and GEO(https://www.ncbi.nlm.nih.gov/geo/), which contained 984 samples from GSE37642 (17), 151 samples from TCGA-LAML, and 304 samples from GSE10358 (18). We then adopted the following criteria to further screen the samples: 1, The tumor primary site of all samples should be bone marrow or peripheral blood. 2, All samples should have complete RNA-seq data and clinical information. 3, All samples shall have complete survival information. After screening, we included 367 samples from GSE37642-GPL96 as our training set, 132 TCGA-LAML samples and 91 GSE10358-GPL570 samples as test set, totaling 590 samples. In addition, the GSE114868 (19) and GSE149237 datasets were downloaded from the GEO database for screening genes that were statistically different (|logFC>1| and p<0.05) between healthy donors and AML patients for subsequent screening. Preprocessing of the data is shown in **Supplementary Figure S1A**.

### 2.2 Lysosome-related gene sets

A total of 191 lysosome-associated genes from five gene sets were obtained by searching the MsigDB database(https://www.gsea-msigdb.org/gsea/msigdb) with the keyword lysosome, 169 genes were extracted from the expression matrix of the training set GSE37642-GPL96 for subsequent analysis, and the specific gene sets and genes are provided in **Supplementary Table 1**.

### 2.3 Consensus unsupervised clustering

We extracted the expression of 169 lysosome genes from the training set GSE37642-GPL96, and obtained the sample clustering information by repeating the calculation 1000 times using the R package “ConsensusClusterPlus”. The differences were initially evaluated by principal component analysis (PCA) and Kaplan-Meier (KM) survival curves, and the expression of genes in different clusters was represented by heatmaps. For secondary clustering, we obtained 87 genes that differed between the two lysosomal clusters and between healthy donors and AML patients by taking the intersection of DEGs from between the two clusters and differential genes from GSE114868 and GSE149237 respectively, and subsequently obtained the results of the secondary clustering of the samples using the same method.

### 2.4 Differential analysis of gene expression, PPI and enrichment analysis

According to the unsupervised consensus clustering, we divided the test set into different clusters, and analyzed the differential genes between the two clusters using the R package “limma” (|logFC>0|p<0.05) (20), and represented them as volcano plot. We obtained 646 differential genes, exported the network through the string (https://cn.string-db.org/), imported it into Cytoscape_v3.8.0, and selected the top30 nodes to obtain the protein-protein interaction(PPI) network after calculating the degree by cytohubba. Gene Ontology (GO) enrichment analysis and Kyoto Encyclopedia of Genes and Genomes functional enrichment analysis of differentially expressed genes using the R package “clusterProfiler” (21).

### 2.5 Immunoinfiltration analysis

The ESTIMATE and CIBERSORT scores were computed using the R package “IOBR” (22). The marker genes of immune cells were sourced from the TISIDB database(http://cis.hku.hk/TISIDB/), and the immune cell enrichment scores were obtained by single-sample gene enrichment score estimation (ssGSEA) analysis with the R package “GSVA” before comparing immune cell infiltration between clusters (23). Immune checkpoint gene set from ref (24).

### 2.6 Construction and validation of a prognostic model

For the 87 DEGs screened, 26 genes were obtained by univariate cox regression (p<0.05), 6 genes were screened by stepwise multivariable cox regression (p<0.05), lasso regression was performed to prevent overfitting, and finally, lysosome-related gene scoring models were constructed according to the following formulae,


$$\;Risk\;Score=\sum_{x=1}^{n}\left(Gene_{x}\times coef_{x}\right)$$

$Gene_{x}$ is the gene expression, $coef_{x}$ is the coefficient of this gene. In accordance with the median value, the dataset is stratified into High-risk and Low-risk groups. The receiver operating characteristic (ROC) curve for the first, third, and fifth year between the High-risk and Low-risk groups were analyzed using the R package “timeROC”. The R packages “regplot” and “rms” were used to produce nomogram and calibration curves. TCGA-LAML, GSE10358-GPL570 were used as test sets and the same calculations were performed.

### 2.7 Prognostic modeling and immunotherapy response

Tumor Immune Dysfunction and Exclusion(TIDE) score was calculated from the website (http://tide.dfci.harvard.edu/), then group comparisons are made by R. The immune infiltration score and the abundance of immune cells were calculated using the R package “IOBR” before group comparisons were made.

### 2.8 Drug sensitivity

The drug sensitivity of the expression matrix of the training set was calculated using the R package “pRRophetic” (25), compared in R according to the grouping information, and finally presented in a box plot.

### 2.9 Cell culture

Cell lines HS-5, KG-1a, HL-60, NB4, U937, and PBMC were obtained from American Type Culture Collection (ATCC), and OCI-AML2 was obtained from the German Collection of Microorganisms and Cell Cultures. KG-1a, HL-60, NB4, and U937 cells were cultured in RPMI-1640 medium (Gibco, USA), while HS-5 was cultured in DMEM (Gibco, USA). OCI-AML2 was cultured in MEM-α medium (Gibco, USA). The media used above contained 10% fetal bovine serum (FBS, USA) and 1% penicillin-streptomycin (Beyotime, Shanghai, China). The PBMC was not cultured after obtaining but was used directly for RNA extraction.

### 2.10 Real-time quantitative reverse transcription PCR

Total cellular RNA was extracted with TRIzol reagent (Takara, Japan) and then reverse transcribed into cDNA using PrimeScript™ RT Master Mix (Takara, Japan). RT-qPCR was performed in a CFX Connect™ RT-qPCR System (Bio-Rad, USA) using Hieff® qPCR SYBR Green Master Mix (Yeasen, Shanghai, China). Pre-denaturation was conducted for 5 min at 95°C, followed by cycling with denaturation at 95°C for 10 s, annealing at 58°C for 30 s, and extension at 72°C for 30 s, repeated for a total of 40 cycles. Up to 40 cycles without results were counted as the maximum of 50 cycles. The relative expression values of six genes in different cell lines were calculated using the method of 2^^-ΔΔCt^, with GAPDH and PBMC used as reference, respectively. The experiments were repeated three times to obtain the data. All primer sequences, synthesized by Sangon Biotech (Shanghai, China), are shown in **Supplementary Table 2**.

### 2.11 Research flowchart

The flow chart for this research is placed in **Supplementary Figure S1B**.

### 2.12 Statistical analysis

Statistical analysis of all data was performed through R (R-4.3.1).t test and Kruskal-Wallis test were used for comparison of two and more groups, respectively. log-rank test was used to evaluate the significance of statistical differences. Where p< 0.05 was considered statistically significant. * p< 0.05; ** p< 0.01; *** p< 0.001.

## 3 Results

### 3.1 Lysosomal subcluster

**Figure 1:**
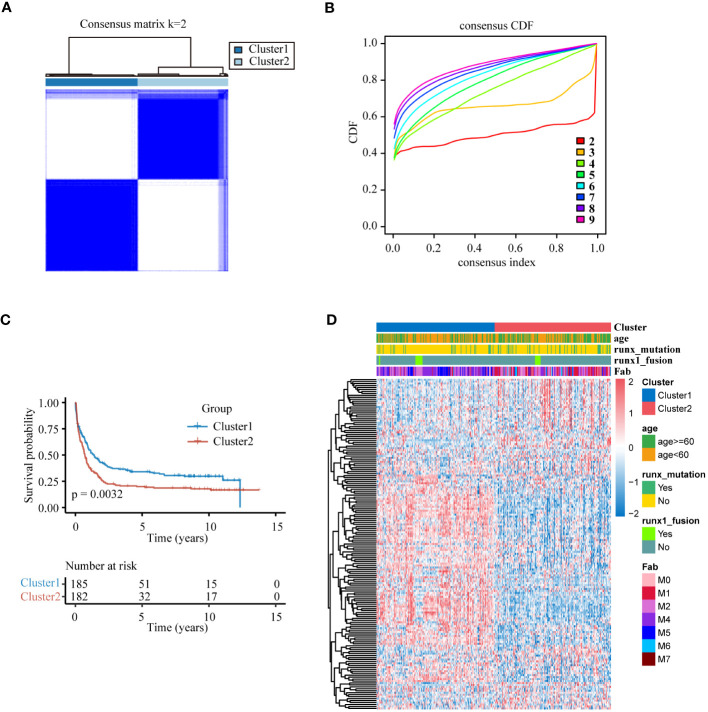
Lysosomal genes clustered for AML patients **(A)** Matrix heatmap at k=2. **(B)** Cumulative distribution function (CDF) for different k values. **(C)** KM survival analysis curves between two clusters at K=2. **(D)** Heat map distribution of lysosomal genes in the training set.

To investigate whether lysosomal genes exhibit specific expression patterns in AML, we employed unsupervised consensus clustering to categorize 367 AML samples. The most obvious expression variations were detected when k=2, resulting in the split of the training set AML samples into two subclusters. Cluster1 (n=185) and Cluster2 (n=182) (**Figures 1A, B**, **Supplementary Figure S2A**). The results of PCA indicated a significant differentiation in gene expression between the two subclusters (**Supplementary Figure S2B**). Based on this, the KM curve suggested a noteworthy survival difference between the distinct subclusters, with the overall survival (OS) time of patients in Cluster1 significantly prolonged compared to Cluster2 (**Figure 1C**). Moreover, patients with the runx1-mutation had a significantly higher representation in Cluster 2, and most lysosomal genes exhibited lower expression in Cluster2 (**Figure 1D**).

### 3.2 Molecular expression and biological processes among lysosomal subclusters

**Figure 2:**
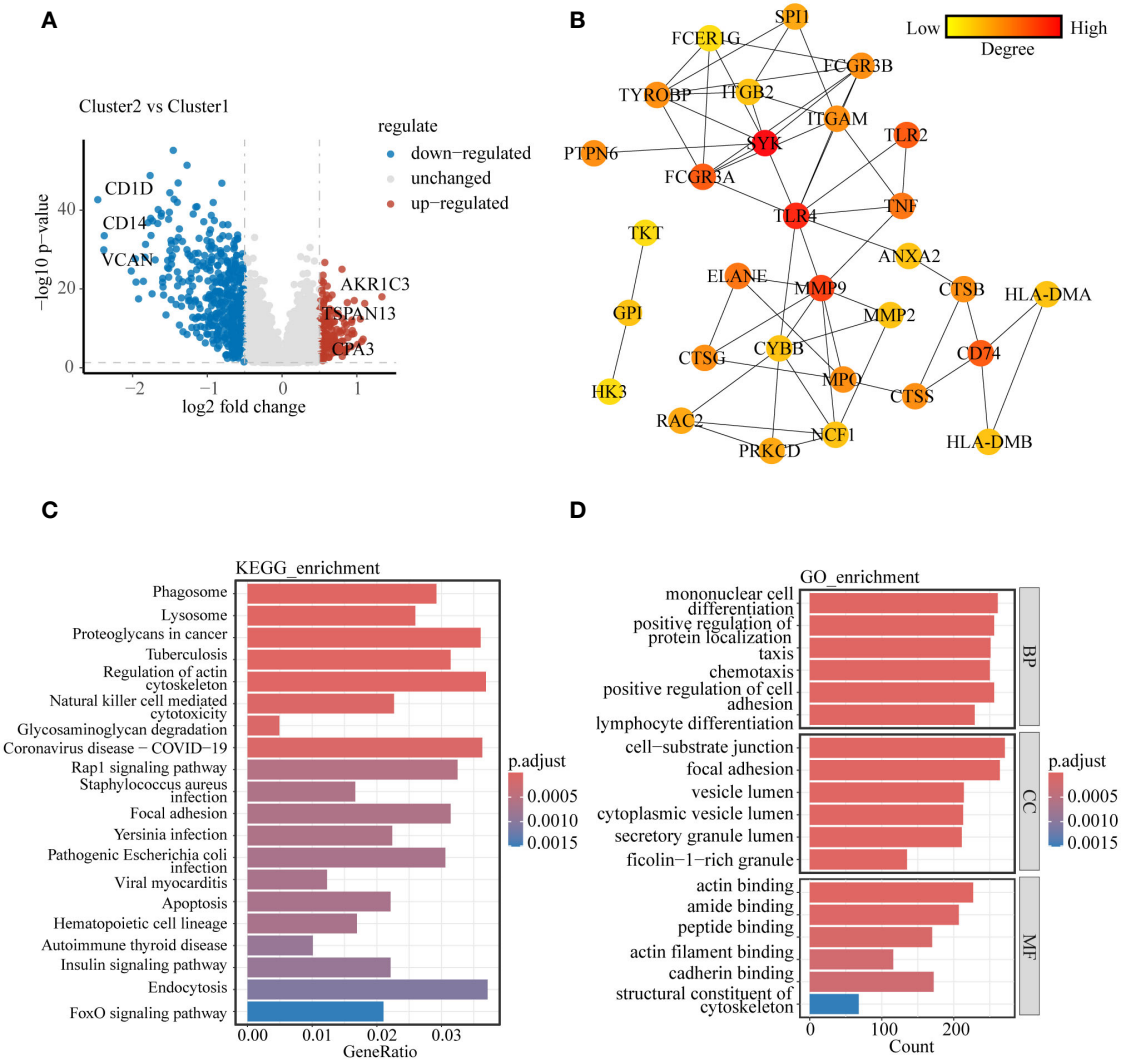
Molecular and biological processes differences between lysosomal clusters **(A)** Volcano diagram showing molecular differences between clusters. **(B)** PPI network was constructed to uncover core genes in DEGs. **(C)** KEGG enrichment analysis and **(D)** GO enrichment analysis to explain the biological processes involved in DEGs between clusters.

To delve further into the distinctions between these two subclusters, We utilized the R package “limma” to analyze the genes responsible for these differences, resulting in the identification of 672 differentially expressed genes (DEGs) (|logFC| > 0.5 and p< 0.05), with 166 up-regulated and 480 down-regulated (**Figure 2A**). To identify the core genes among these DEGs, we computed the top 30-degree core genes by Cytoscape, revealing two modules centered on spleen tyrosine kinase (SYK) and toll-like receptor 4 (TLR4), both tightly linked to the regulation of immune function (**Figure 2B**). These DEGs were enriched into lysosomes, cellular vesicles, immune cell functions, apoptosis, and some signaling pathways analyzed by KEGG enrichment (**Figure 2C**). GO enrichment analysis demonstrated the involvement of DEGs in cytoskeletal regulation, vesicle membrane composition, and other aspects (**Figure 2D**). Similar results were obtained by enrichment analysis of up- and down-regulated genes separately (**Supplementary Figures S3A–D**). These findings tentatively corroborated the subcluster results of our study.

### 3.3 Immune infiltration between lysosomal subclusters

**Figure 3:**
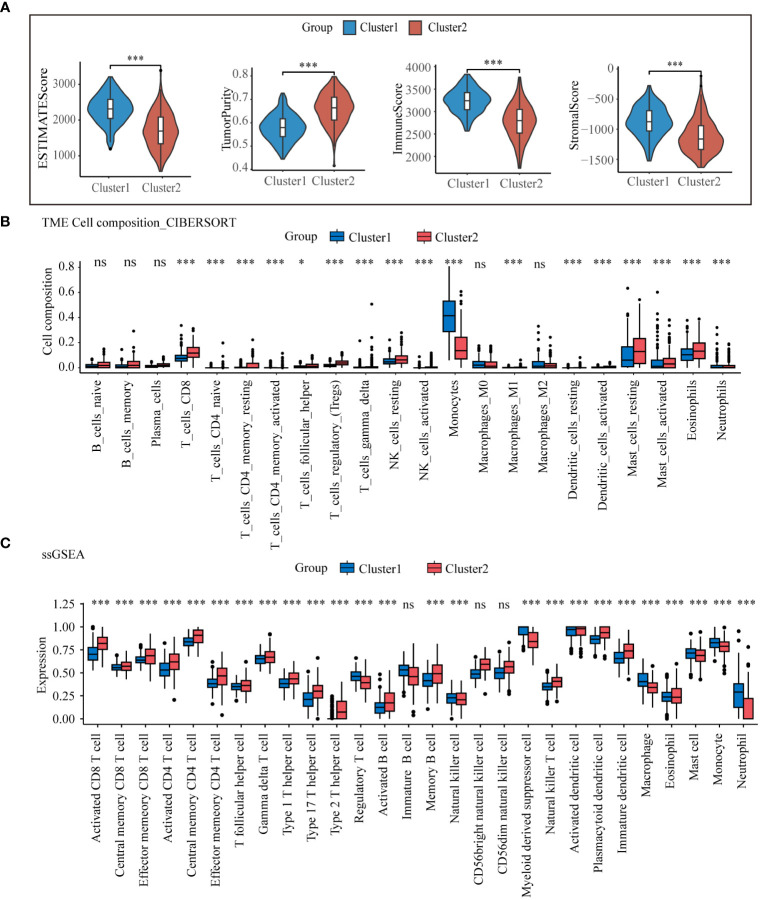
Tumor microenvironmental analysis of lysosomal clusters Estimate analysis comparing **(A)** estimate score, **(B)** CIBERSORT calculated the relative compositional abundance of 22 immune cells. **(C)** ssGSEA calculated the relative abundance of immune cells in 28. ns, non-significant; * p< 0.05; *** p< 0.001.

The results from the previous PPI core gene and enrichment analyses revealed significant differences in immunomodulatory pathways between the two lysosomal subclusters. To gain a deeper understanding of the immune microenvironmental distinctions between the subclusters, we computed ESTIMATE scores for both subclusters using the R package “IOBR”. The ESTIMATE scores indicated that in cluster1, there was greater immune cell infiltration and lower tumor purity compared to cluster2 (**Figure 3A**). The infiltration of these immune cells may play an anti-tumor role. The relative abundance of selected immune cells was further estimated for all training set samples using CIBERSORT and ssGSEA (**Figures 3B, C**). The results demonstrated predominant enrichment of monocytes, macrophages, and neutrophils in cluster1, while T cell subsets such as CD8 and CD4+ T cells were enriched in cluster2. The tumor immune response is influenced by the crosstalk between tumor cells, immune cells, and immune molecules. According to the expression of immune checkpoint genes (**Supplementary Figure S4**), partial immune checkpoint genes were significantly different between the two groups.CD86, whose expression was significantly lower in cluster2 than in cluster1, exerts anti-tumor effects by binding to CD28, inducing T cells to continue proliferating and differentiating into effector T cells (26). The above results indicate significant differences in the immune microenvironment of the two lysosomal subclusters, with cluster1 exhibiting stronger immune cell infiltration and a more robust immune response than cluster2. These differences offer potential therapeutic targets for achieving individualized treatment.

### 3.4 Secondary clustering

**Figure 4:**
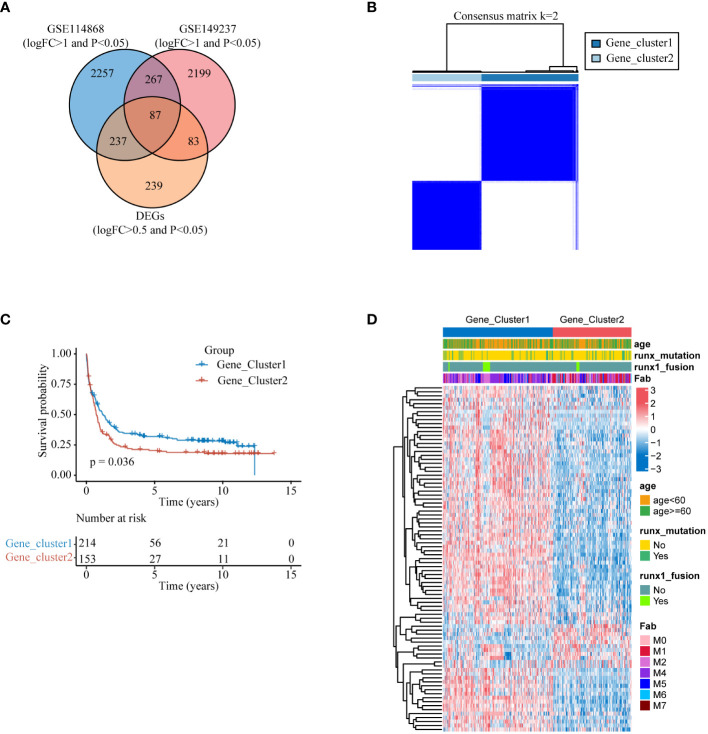
Secondary clustering **(A)** DEGs that vary among lysosomal isoforms and also exhibit differences in expression between AML patients and healthy individuals. **(B)** Heat map of the consensus matrix of the sample at k=2. **(C)** KM survival analysis curves between two gene subtypes at K=2. **(D)** Heatmap of the expression of 87 differential genes between the two gene subtypes.

To enhance integration with clinical diagnosis, we initially identified genes exhibiting expression disparities (|logFC| > 1 and p< 0.05) between AML patients and healthy donors from datasets GSE114868 and GSE149237, respectively. We then intersected this selection with genes from the training set GSE37642-gpl96, which had expression differences (|logFC| > 0.5 and p< 0.05) between the two molecular subtypes, to obtain 84 DEGs (**Figure 4A**). We employed these 87 differentially expressed genes for unsupervised consensus clustering. The clustering results indicated optimal typing at K=2 (**Figure 4B**, **Supplementary Figures S5A–C**), and KM curve revealed that Gene_cluster1 had significantly higher overall survival time than Gene_cluster2 (**Figure 4C**).The heatmap illustrates the expression patterns of the 87 DEGs between the two gene subtypes and their correlation with clinical features (**Figure 4D**).

### 3.5 Construction of a prognostic model for lysosome-related genes

**Figure 5:**
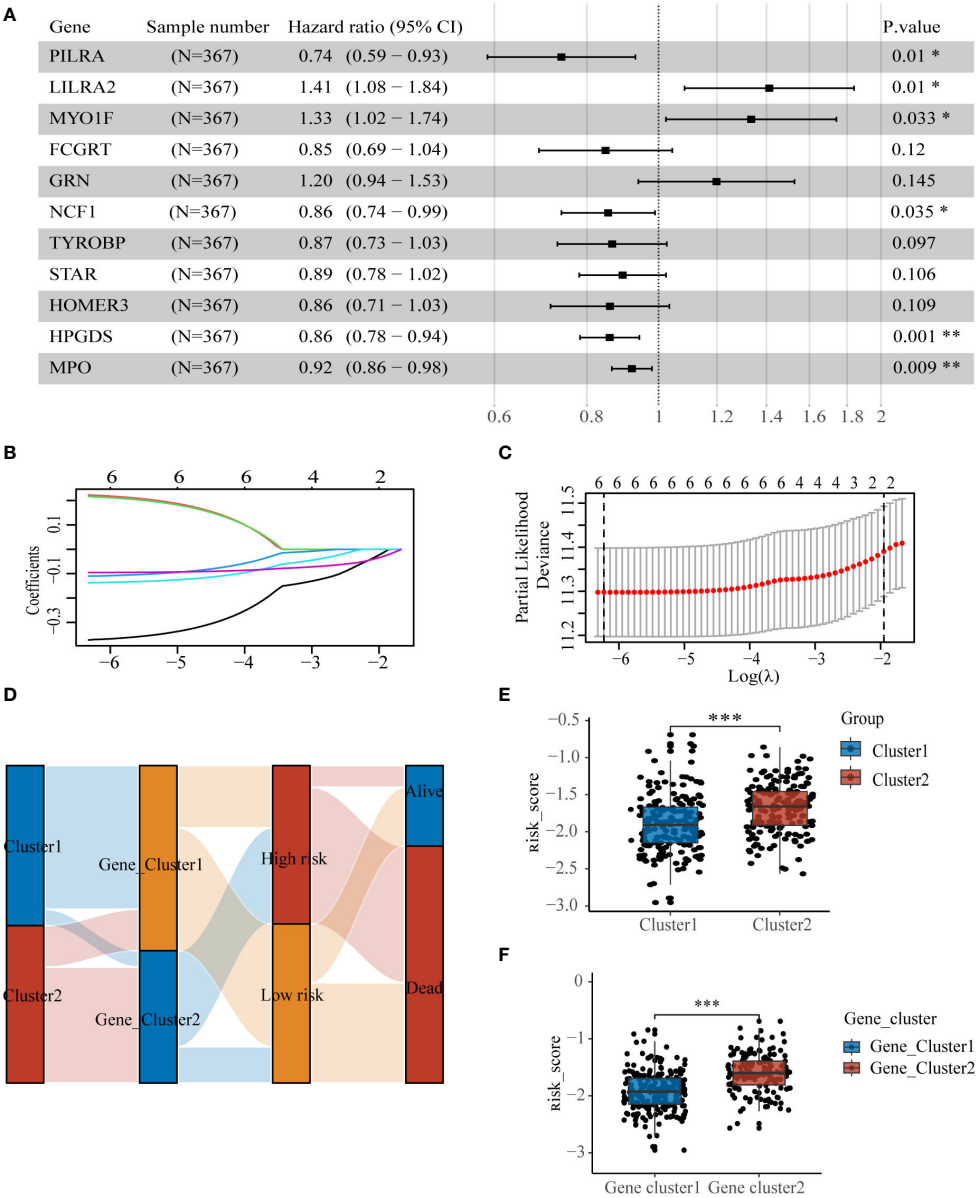
Construction of lysosome-related gene prognostic model **(A)** Multivariate regression results screened 6 DEGs, **(B, C)** lasso regression screened 6 DEGs for construction of prognostic model. **(D)** Sankey diagrams clearly show the distribution of patients among different subgroups and the outcome of **(E)** molecular subtypes and **(F)** risk scores for genetic subtypes. * p< 0.05; ** p< 0.01; *** p< 0.001.


To identify genes influencing prognosis between the two lysosomal subclusters, we conducted univariate cox regression (p< 0.05) on the 87 DEGs obtained from the intersection (**Supplementary Figure S6**). We identified 26 DEGs significantly impacting prognosis. Further screening was performed using multivariate cox regression (p< 0.05) (**Figure 5A**). To prevent overfitting, we employed lasso regression and constructed a prognostic model comprising 6 genes (**Figures 5B, C**). The sample’s risk score was computed based on the formula:

$$\begin{array}{l} Risk\;Score=\;\;expression\left(PILRA\right)\times coef\left(-0.370\right)+expression\left(LILRA2\right)\times coef\left(0.221\right)\\ \;+expression\left(MYO1F\right)\times coef\left(0.214\right)+expression\left(NCF1\right)\times coef\left(0.110\right)\\ \;+expression\left(HPGDS\right)\times coef\left(-0.137\right)+expression\left(MPO\right)\times coef\left(-0.095\right) \end{array}$$

The samples from the dataset GSE37642-GPL96 were divided into two groups based on the median values of the risk scores. The sankey diagram illustrates the association between several subtypes and patient survival outcomes(**Figure 5D**). Cluster2 and Gene_cluster2, associated with worse prognosis, exhibited significantly higher risk scores than Cluster1 and Gene_cluster1(**Figures 5E, F**).

### 3.6 Validation of the lysosome related-genes prognostic model

**Figure 6:**
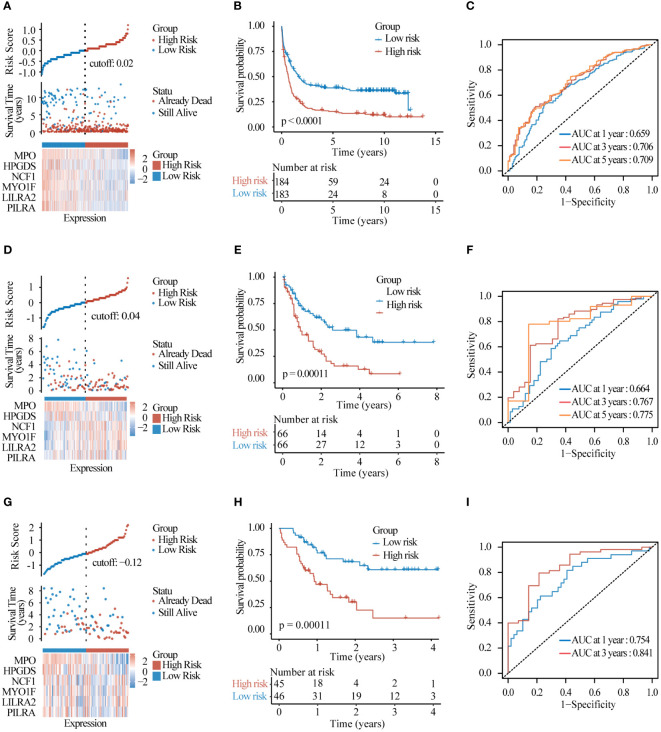
Model evaluation and validation **(A)** Risk factor distribution plots and **(B)** KM survival curves and **(C)** timeROC curves assessing the predictive accuracy of the risk model in the test set GSE37642-GPL96. **(D–F)** The same was performed in test set TCGA-LAML and **(G–I)** test set GSE10358-GPL570.

To test the predictive effect of lysosomal related-genes prognostic model on the prognosis of AML patients, we first examined the distribution of risk scores of patients in the training set by ggrisk (**Figure 6A**), and the patients with greater risk scores had higher risk of death. The results of the KM curves hinted to the fact that patients in the high-risk group had a much lower OS than those in the low-risk group (**Figure 6B**), and the 1-, 3-,and 5-year AUC of ROC were 0.659,0.706,0.709 respectively (**Figure 6C**). These results demonstrated the good performance of the lysosomal risk score model in predicting the survival of AML patients. Further, we observed similar results in the test set data TCGA-LAML (**Figures 6D–F**) and GSE10358-GPL570 (**Figures 6G–I**). As the risk score increases, the risk of patient death increases, which provides an important basis for identifying high-risk patients. These results suggest that our lysosomal prognostic model can be used as a reliable survival predictor, which can help to more accurately stratify patients and assess prognosis.

### 3.7 Nomogram

We plotted the nomogram in conjunction with other clinical characteristics such as age, FAB typing for the purpose of further evaluating the model, patients with lower risk scores and younger age had better prognosis (**Supplementary Figure S7A**). The calibration curve showed the agreement between our prognostic model and real events (**Supplementary Figure S7B**). Similar results were observed in the test set data TCGA-LAML (**Supplementary Figures S7C, D**) and GSE10358-GPL570 (**Supplementary Figures S7E, F**). In addition, expanding the sample size and wider data validation are more helpful to strengthen the predictive power and clinical application value of risk lysosomal risk score in different populations.

### 3.8 Lysosomal scores predict immunotherapy effects

**Figure 7:**
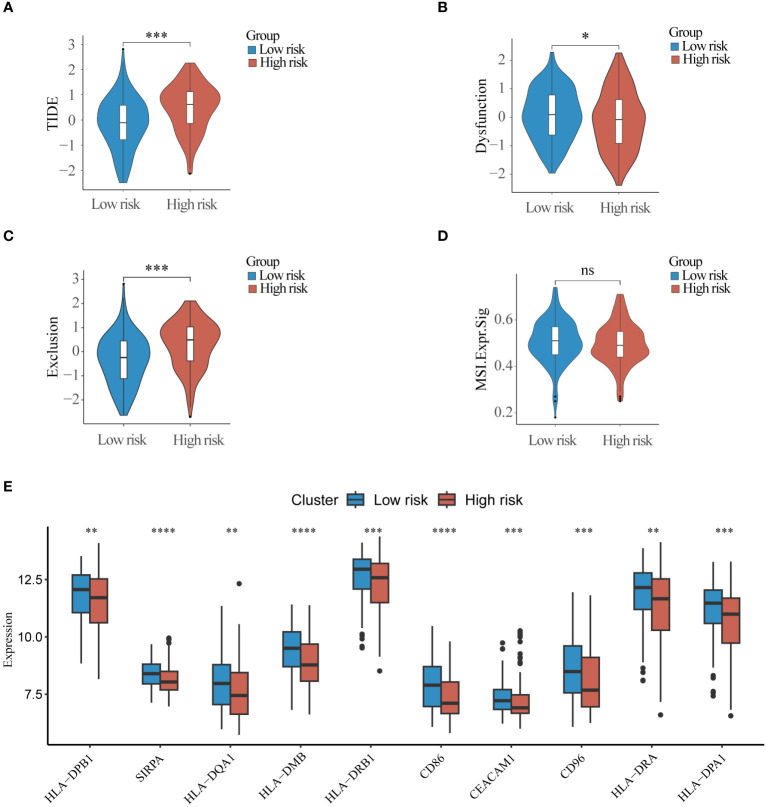
Immunotherapy response differences between high and low-risk groups **(A)** TIDE score, **(B)** Dysfunction score **(C)** Exclusion score and **(D)** Microsatellite instability score was used to compare differences in response to immunotherapy between high and low risk groups. **(E)** Top10 immune checkpoint molecules differentially expressed in high and low risk groups. ns, non-significant; * p< 0.05; ** p< 0.01; *** p< 0.001.

TIDE scores were calculated for the purpose of evaluating the role of risk scores in immunotherapy, and the TIDE scores of the high-risk group were significantly higher than those of the low-risk group (**Figures 7A–D**), suggesting that the high-risk group may be more susceptible to immune escape. Some immune checkpoints associated with MHC-II molecules were significantly less expressed in the high-risk group compared to the low-risk group(**Figure 7E**). The results of the ESTIMATE scores showed that immune infiltration was significantly stronger in the low-risk group than in the high-risk group (**Supplementary Figures S8A–D**), and the infiltration abundance of most immune cells was significantly with the high-risk group (**Supplementary Figure S8E**). These results suggest that there is a significant difference between the high-risk and low-risk groups in terms of response to immunotherapy in the training set data, and that the scoring model can effectively guide immunotherapy.

### 3.9 Drug sensitivity

**Figure 8:**
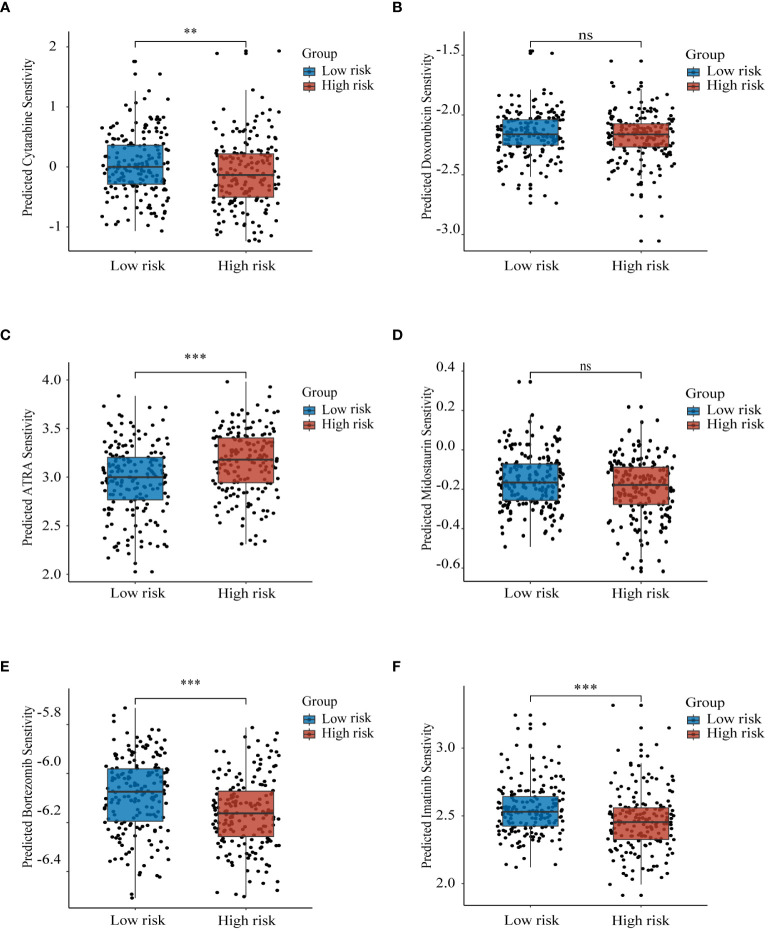
Prognostic model to guide drug therapy Sensitivity of high and low risk groups to different drugs in the training set GSE37642-GPL96 samples with **(A)** cytarabine, **(B)** doxorubicin, **(C)** ATRA, **(D)** midostaurin, **(E)** bortezomib, and **(F)** imatinib. ns, non-significant; ** p< 0.01; *** p< 0.001.

We analyzed the sensitivity of the training set samples to several drugs by using the R package “pRRophetic”. As compared with the low-risk group, the high-risk group was more sensitive to cytarabine (**Figure 8A**), ATRA (**Figure 8C**), imatinib (**Figure 8E**), and bortezomib (**Figure 8F**). There was no significant difference between the two groups in sensitivity to doxorubicin (**Figure 8B**) and midostaurin (**Figure 8D**). The above results provide important reference evidence for clinical treatment.

### 3.10 Validation of gene expression

**Figure 9:**
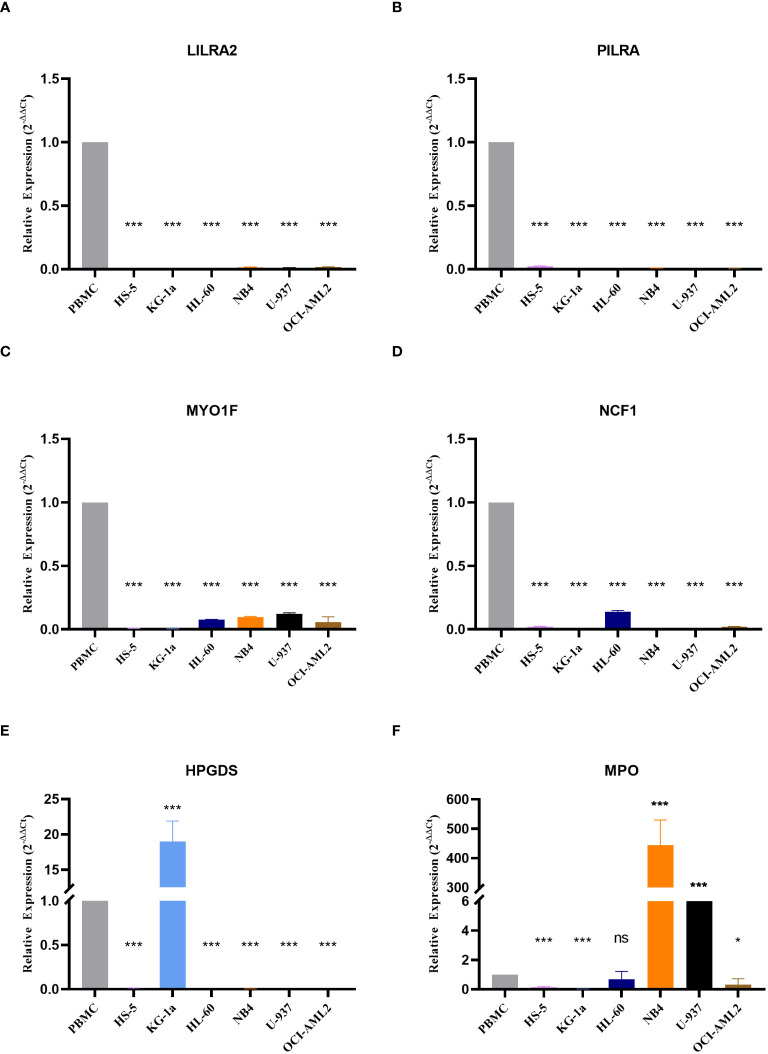
Expression of the six genes modeled Expression values of the six genes used to construct the model in normal and AML cell lines are depicted, including **(A)** LILRA2, **(B)** PILRA, **(C)** MYO1F, **(D)** NCF1, **(E)** HPGDS, and **(F)** MPO. ns, non-significant; * p< 0.05; *** p< 0.001.

To validate the expression of the six genes utilized in model construction, we initially selected dataset GSE114868 to compare gene expression between healthy donors and AML patients. The results revealed significant downregulation of PILRA, LILRA2, MYO1F, and NCF1 in AML, while HPGDS and MPO exhibited heightened expression levels (**Supplementary Figure S9**). Subsequently, we corroborated these findings using cell lines. Consistent with dataset GSE114868, we observed notable reductions in PILRA, LILRA2, MYO1F, and NCF1 expression, alongside significant elevations in HPGDS and MPO expression in AML cell lines compared to normal cells (**Figures 9A–D**). Notably, HPGDS was predominantly overexpressed in KG-1a cells, with relatively low expression in other AML cell lines (**Figure 9E**), while MPO expression in NB4 and U937 cells surpassed that of normal cells by more than 50-fold (**Figure 9F**).

## 4 Discussion

The current risk assessment for AML relies predominantly on the identification of genetic traits through gene sequencing and other methods for risk classification. However, this approach is hindered by issues such as prolonged duration, reliance on a single method, and low accuracy (27, 28). In contemporary cancer research, molecular classification and prognostic modeling are increasingly turning their focus toward the intricate roles played by subcellular entities, or organelles. While existing risk models have predominantly centered on mitochondria, particularly in the context of cancer cells undergoing metabolic reprogramming, the lysosome, despite its equally pivotal role in cancer cell metabolism, has been relatively neglected (29–32).

Our study has developed molecular subtype and prognosis-related risk models in AML centered on lysosomal-related genes. This is the first model constructed based on lysosomal genes in AML. In this study, disparities in lysosomal gene expression were instrumental in classifying AML patients into distinct molecular subtypes, which differed significantly in terms of patient prognosis, molecular expression, and immune infiltration. However, unlike other similar studies (33), we refrained from conducting prognostic screening of the gene set prior to molecular subtyping. While this approach may diminish the model’s prognostic predictive capacity, it facilitates the identification of other essential biological features beyond prognosis.

Our PPI results reveal that the pivotal differential genes distinguishing between the two lysosomal isoforms are SYK and TLR4. SYK, a non-receptor tyrosine kinase, has garnered significant attention in numerous studies as a promising target for hematologic malignancies and inflammation-related diseases (34). In prior research, SYK’s pro-carcinogenic mechanism has been proposed to regulate the activation of associated pathways through signal transduction, thereby promoting AML cell survival and drug resistance (34, 35). Our study suggests, for the first time, a connection between SYK and lysosomes in AML, a proposition supported by several studies in non-tumor cells (36, 37). Exploring this connection may bring new insights into SYK inhibitor resistance. Toll-like receptor 4 (TLR4) belongs to the Toll-like receptor (TLR) family, plays a pivotal role in pathogen recognition and innate immune activation (38). TLR4 responds to stimulation to activate signaling pathways, such as AMPK, and also regulates the tumor microenvironment, thereby influencing tumor progression (39, 40). A connection between lysosomes and TLR4 has been unveiled, with lysosomes serving as a site for TLR4 degradation (41). However, whether TLR4 modulates lysosomal function remains elusive, and our findings offer additional reference evidence for this avenue of investigation, more extensive studies are warranted to delve into the TLR4-lysosomal connection and its precise mechanism.

We constructed prognostic model comprising 6 genes (PILRA, LILRA2, MYO1F, NCF1, HPGDS, MPO) and categorized patients into high- and low-risk groups. The distribution of risk scores between clusters and Gene_clusters provided a preliminary indication of the accuracy of the scoring model. The results from risk factor distribution plots, KM curves, and TimeROC demonstrated that the high-risk group had worse prognostic outcomes. Nomograms and calibration curves further illustrated the reliability of our model, and these results were validated in two other distinct datasets. Among these genes, MPO, HPGDS, and PILRA were considered favorable prognostic factors. Myeloperoxidase (MPO) regulates inflammatory responses and participates in the regulation of oxidative stress homeostasis (42). It is a common diagnostic marker in hematological neoplasms and aids in differentiating between myeloid and lymphoid lineages in acute leukemias (43). High expression of MPO is correlated with a favorable prognosis in AML patients (44). Hematopoietic prostaglandin d synthase (HPGDS) is an enzyme that catalyzes the isomerization of prostaglandin h2 (PGH2) to prostaglandin d2 (PGD2) (45). It exerts antitumor effects by catalyzing the production of PGD2 (46). Paired immunoglobulin-like type 2 receptor alpha (PILRA) is predominantly expressed on monocytes and macrophages (47) and is involved in the regulation of neutrophil infiltration (48). High expression of PILRA enhances the effect of antitumor immunotherapy (49), but its effects vary in different cancers (50).On the other hand, MYOIF, ILRA2, and NCF1 are considered prognostically unfavorable factors. Studies have demonstrated that MYOIF enhances the adhesion and migration of immune cells (51), promotes M1 polarization of macrophages (52), and in some tumor patients, MYO1F is mutated to form fusion proteins (53, 54), promoting tumorigenesis and progression (55). Activation of LILRA2 inhibits monocyte function and antigen presentation by dendritic cells (56, 57), and high expression of LILRA2 has been associated with a poor tumor prognosis (58). NCF1 encodes a protein that is one of the subunits of NADPH oxidase, and inhibition of NCF1 induces differentiation of APL cells as well as inhibits melanoma cell growth (59, 60). These pieces of evidence strongly support the reliability of our model. However, with the exception of MPO, the above genes have been rarely reported in AML, and follow-up studies are needed to delve deeper into their functions and mechanisms in AML.

Immunotherapy serves as a pivotal therapeutic approach in which lysosomes assume a significant role. Lysosomes facilitate immune evasion by cancer cells through the degradation of crucial proteins, including PD-L1 and MHC- I (61, 62). Only a minute fraction of current AML studies have delved into the influence of lysosomes on immunotherapy (63). Our findings reveal distinct immune responses and variations in the expression of immune checkpoint molecules between high and low-risk groups. Remarkably, multiple immune checkpoint molecules exhibited significant downregulation in the high-risk group, potentially contributing to the observed differences in immunotherapeutic responses (64). Notably, heightened expression of MHC-II class molecules has been consistently linked to favorable prognoses across various tumor types. This link has been confirmed by some studies in AML (65–68). While considerable attention has been devoted to exploring the impact of MHC-II molecules on antitumor immunotherapy, there appears to be a dearth of research investigating the relationship between MHC-II molecules and lysosomes in AML, despite such associations being reported in other disease models (69, 70). Our study might shed light on subsequent lysosome-mediated immunotherapy for AML. Furthermore, our study uncovered lysosome-associated differences in drug sensitivity between high- and low-risk groups, Consistent with this finding, lysosomes have been implicated in conferring drug resistance in cancer cells through mechanisms involving the segregation of drugs within the lysosomal compartment (71).

In conclusion, we have constructed a prognostic model centered on lysosome-related genes for the first time in AML. Our model can effectively assess the prognosis of patients and guide their clinical treatment, which provides new reference evidence for individualized treatment of AML. However, our study also has many limitations. One limitation is that, the study only focused on the association between lysosome-associated mRNAs and AML prognosis, lacking research on non-coding RNAs such as lncRNAs, circRNAs, and tRNAs. Second, external validation of clinical samples is required to ensure the accuracy of the scoring model.

## Data availability statement

The datasets presented in this study can be found in online repositories. The names of the repository/repositories and accession number(s) can be found in the article/**Supplementary Material**.

## Author contributions

PW: Writing – original draft, Writing – review & editing, Conceptualization, Data curation, Formal analysis, Investigation, Methodology, Software, Validation, Visualization. LZ: Project administration, Supervision, Writing – review & editing. LY: Funding acquisition, Writing – review & editing, Resources, Supervision. CS: Conceptualization, Methodology, Writing – review & editing. XS: Data curation, Investigation, Software, Writing – review & editing. SC: Data curation, Investigation, Software, Writing – review & editing. ZZ: Data curation, Investigation, Software, Writing – review & editing. MW: Data curation, Investigation, Software, Writing – review & editing. HZ: Data curation, Investigation, Software, Writing – review & editing. BL: Conceptualization, Funding acquisition, Project administration, Resources, Supervision, Writing – review & editing.
