# Supplementary material for: Lysosome-related genes predict acute myeloid leukemia prognosis and response to immunotherapy
Source: Front Immunol. 2024 May 10;15:1384633. doi: 10.3389/fimmu.2024.1384633 (PMC11117069; doi:10.3389/fimmu.2024.1384633)
Supplement: Supplementary file 2 [file DataSheet_1.docx]

Supplementary Material

# Supplementary Data

Supplementary Material should be uploaded separately on submission. Please include any supplementary data, figures and/or tables.

Supplementary material is not typeset so please ensure that all information is clearly presented, the appropriate caption is included in the file and not in the manuscript, and that the style conforms to the rest of the article.

# Supplementary Figures and Tables

## Supplementary Figures


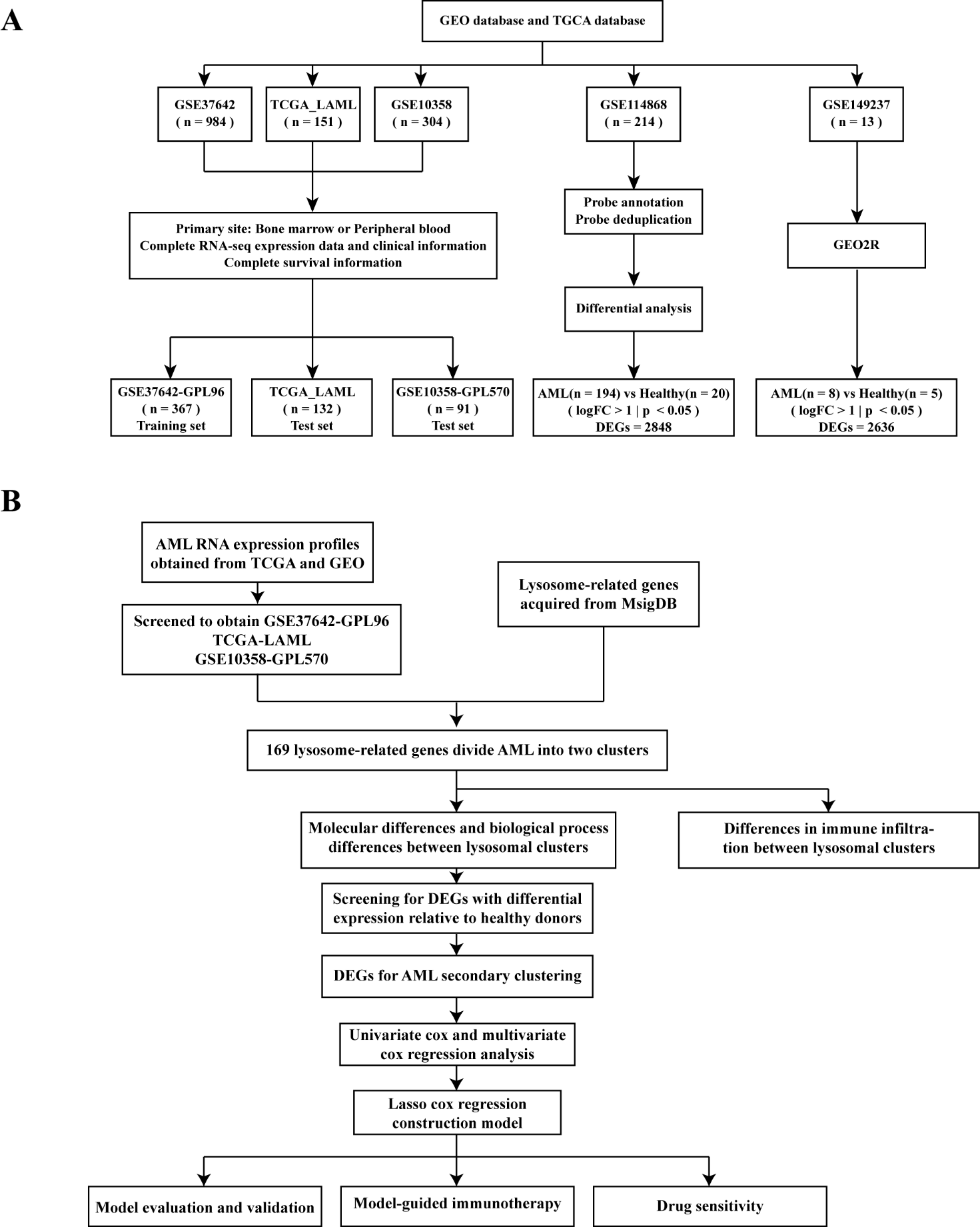


**Supplementary Figure 1.** (A) Flowchart of data preprocessing and (B) flowchart of the whole study.


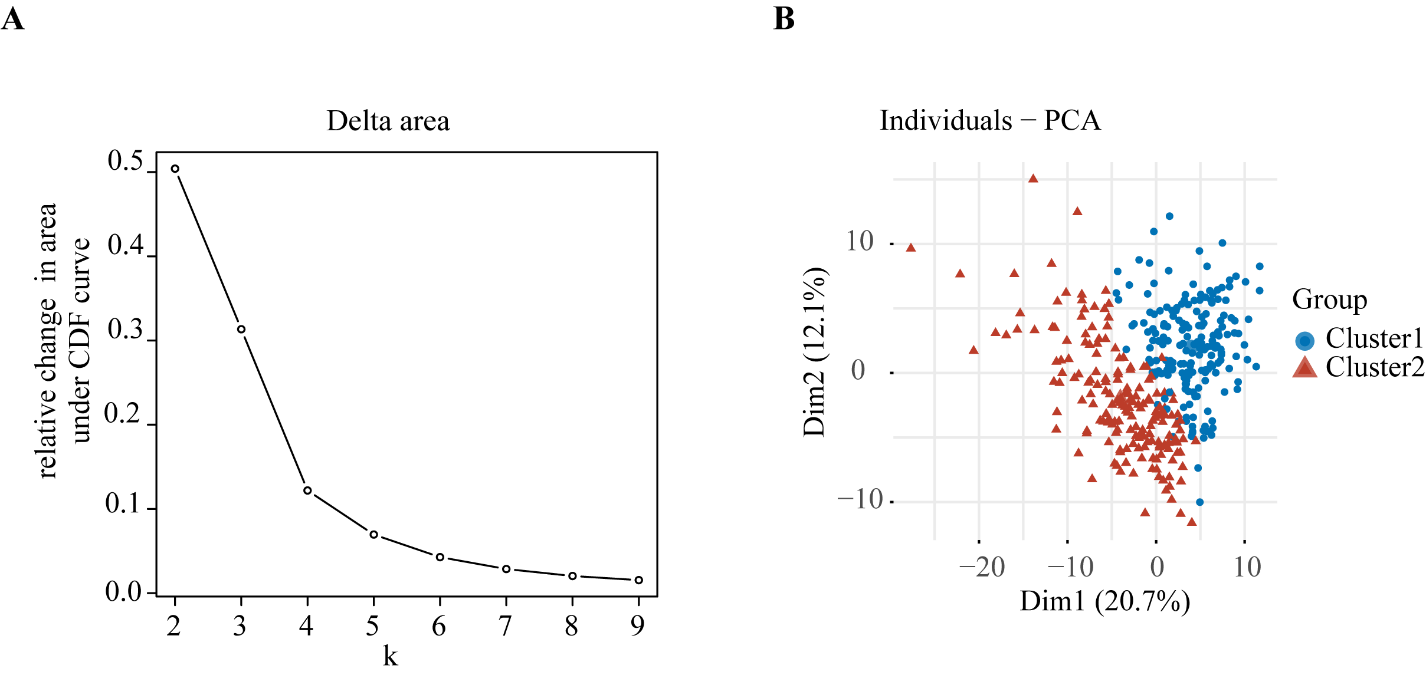


**Supplementary Figure 2.** (A) Relative area under the curve of CDF at different k values. (B) Distribution of samples revealed by PCA at k=2.


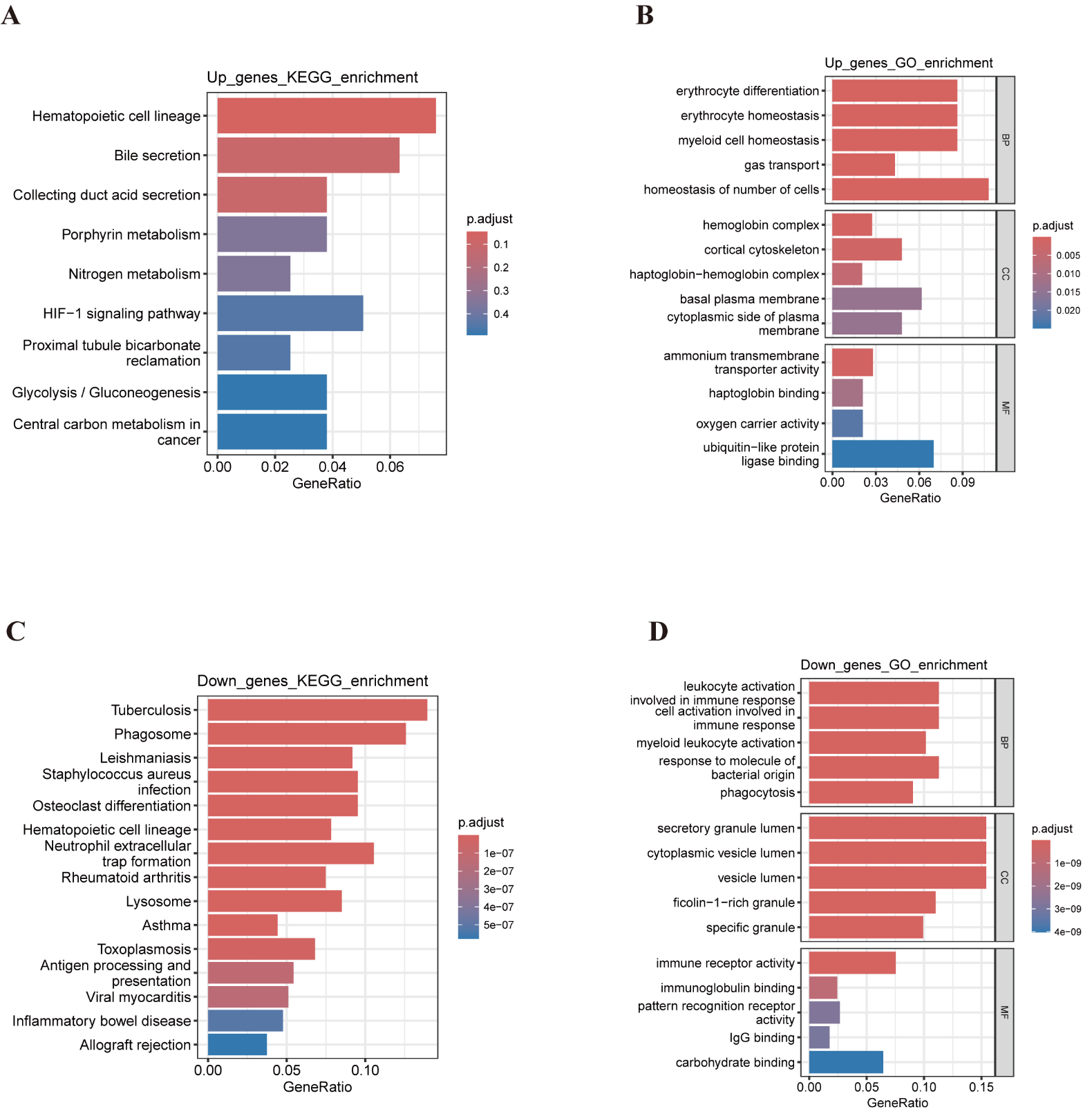


**Supplementary Figure 3. KEGG and GO analyses separately on the up- and down-regulated gene sets** (A) KEGG and (B) GO enrichment analysis of the set of up-regulated genes. (C) KEGG and (D) GO enrichment analysis of the set of down-regulated genes.


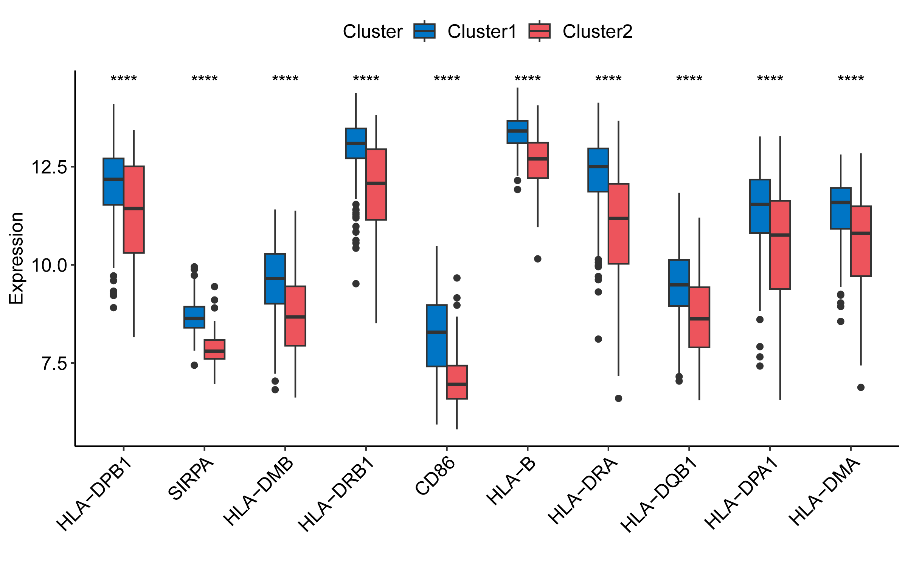


**Supplementary Figure 4.** Top10 immune checkpoint molecules differentially expressed in two clusters.


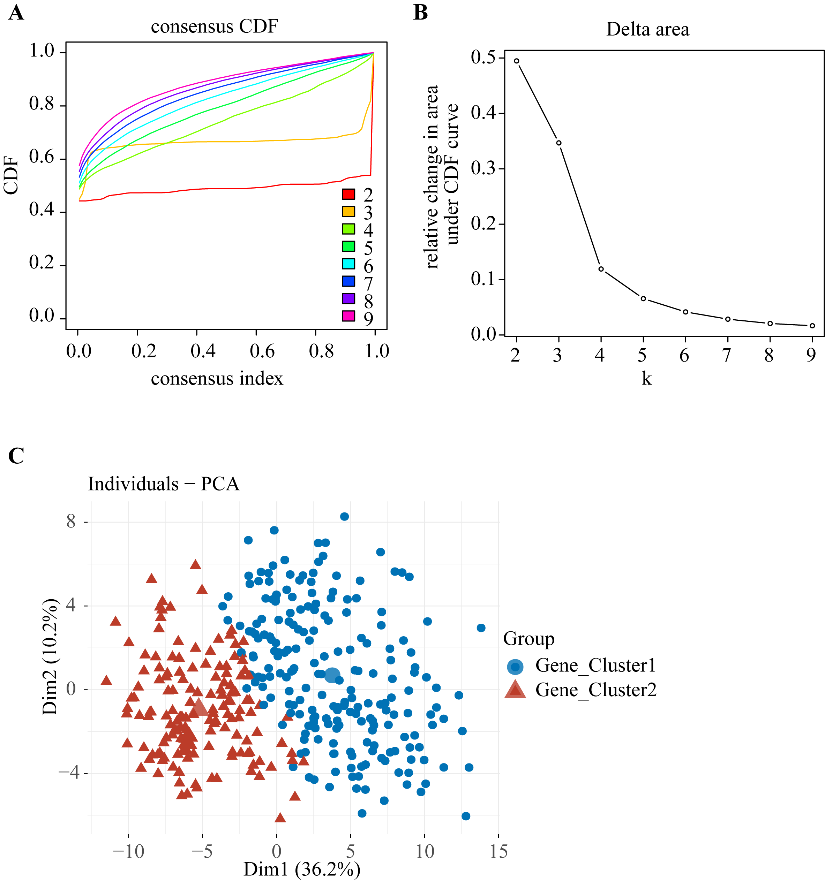


**Supplementary Figure 5.** (A) CDF for different k values. (B) Relative area under the curve of CDF at different k values. (C) Consistency of sample distribution among gene subtypes demonstrated by PCA results


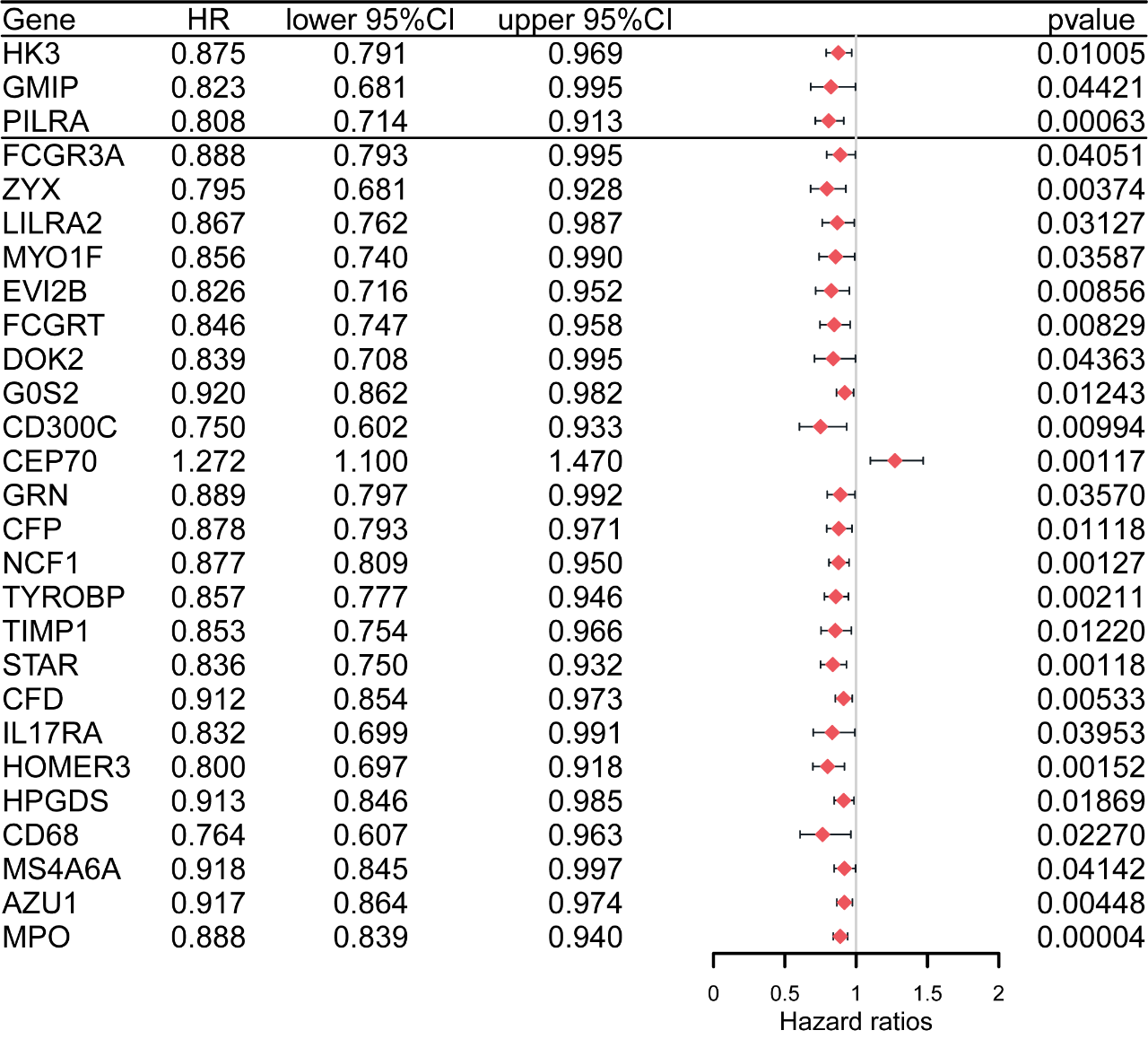


**Supplementary Figure 6.** Univariate Cox regression analysis identified differential genes associated with prognosis.


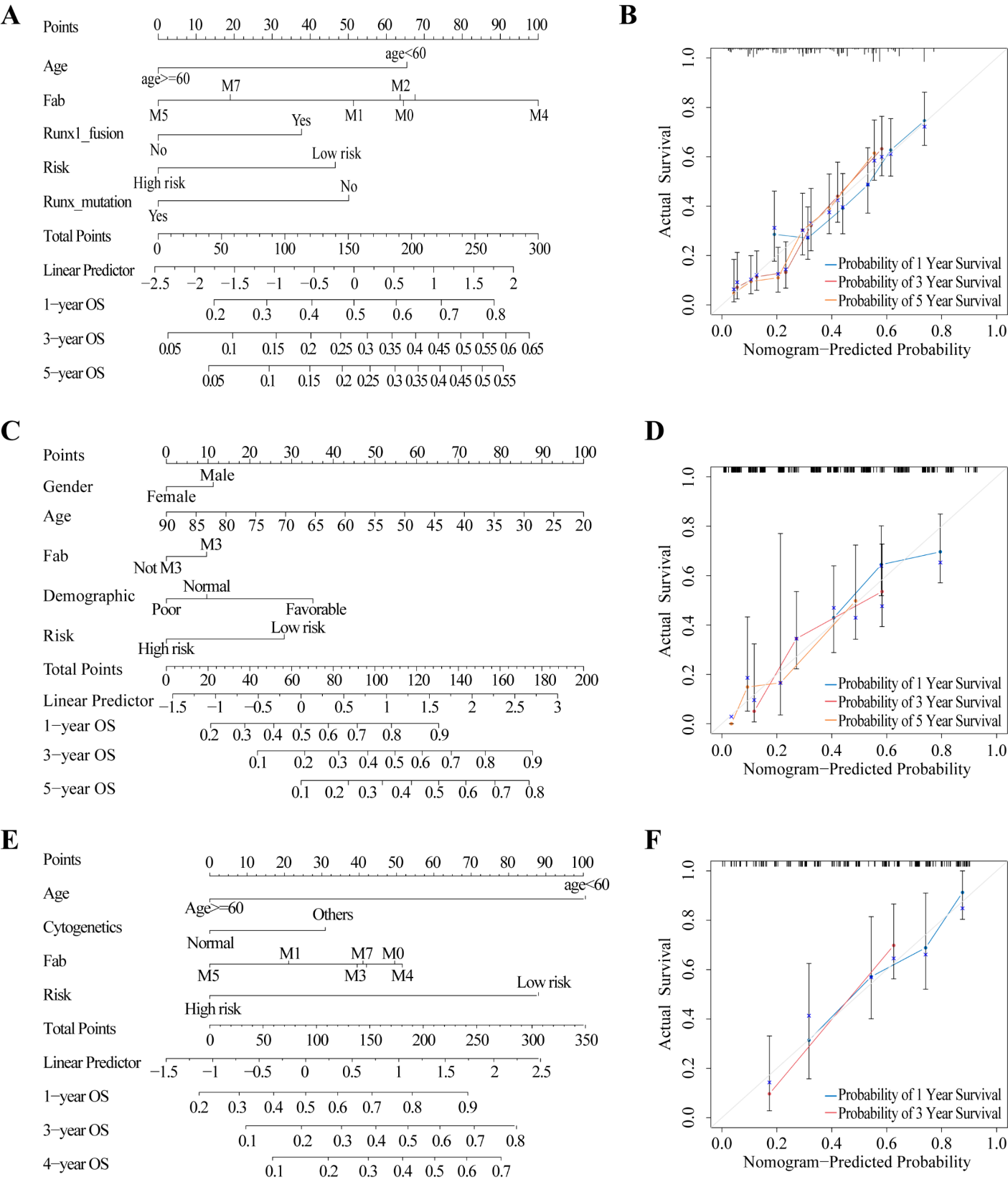


**Supplementary Figure 7. Nomogram and calibration curves** Prognostic models combining different clinical features to draw the (A) nomogram and (B) calibration curves in GSE37642-GPL96. (C)The nomogram and (D) calibration curves of TCGA-LAML. (E,F) The same operation was performed combining the clinical features of GSE10358-GPL570.


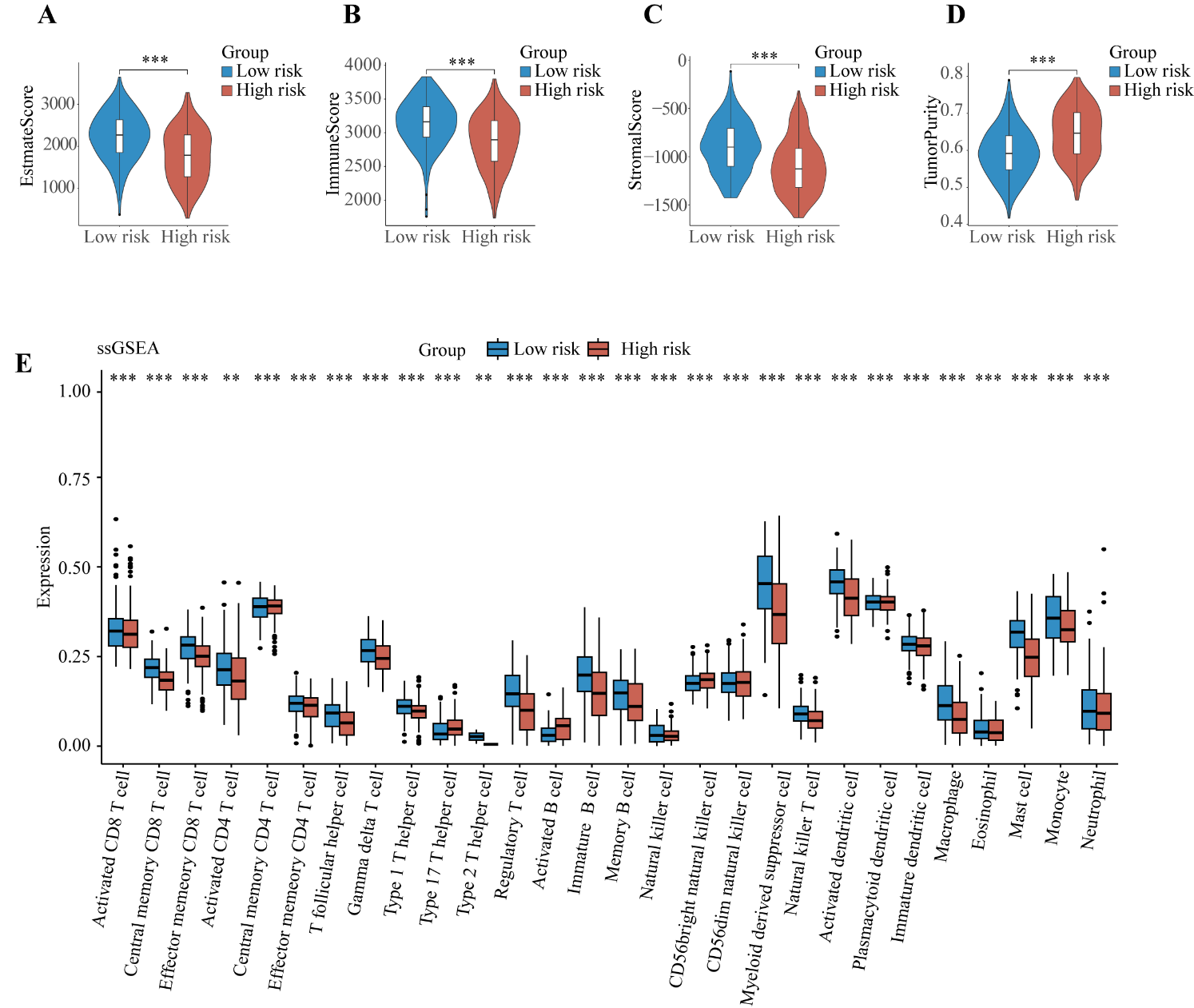


**Supplementary Figure 8. Tumor immune microenvironment in high and low risk groups**

(A) ESTIMATE score, (B) Immunity score, (C) Stroma score and (D) Tumor purity was used to compare immune infiltration between high and low risk groups. (E) The relative abundance of 28 immune cells in the high and low risk groups of the test set was calculated by ssGSEA.


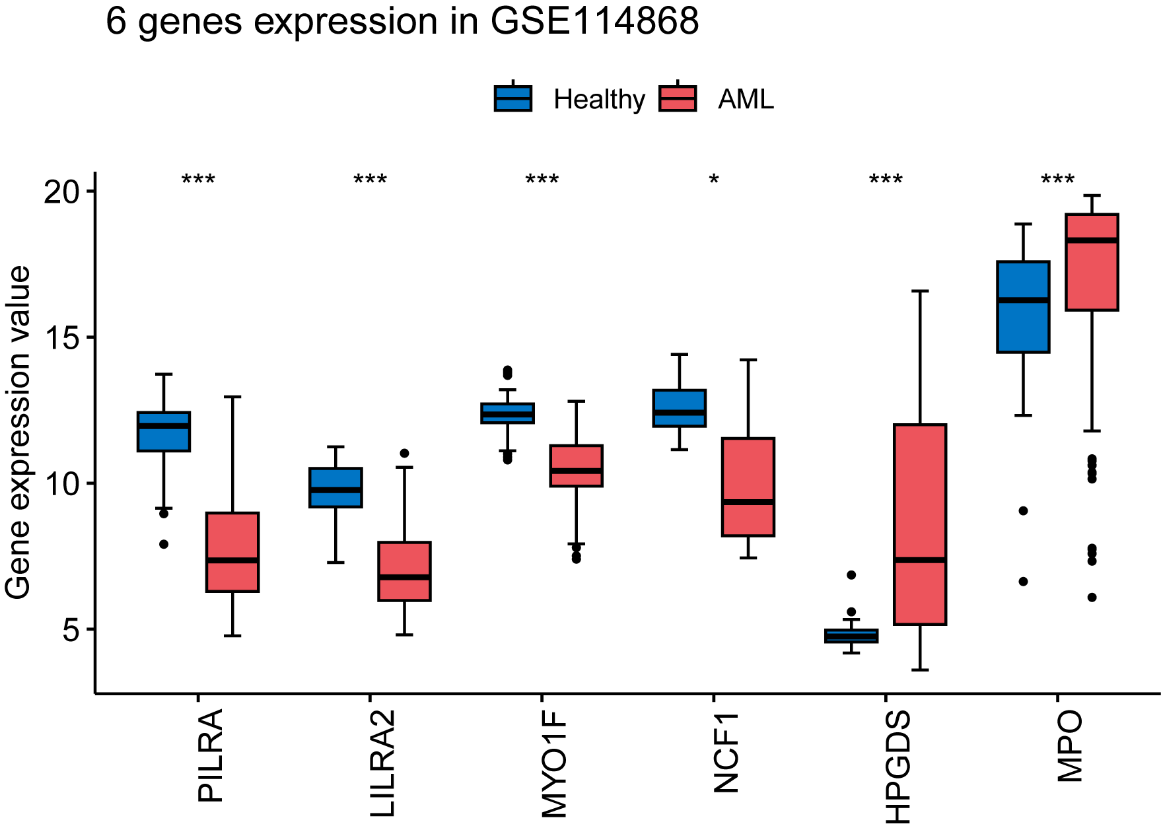


**Supplementary Figure 9.** Expression values of six genes

## Supplementary Tables

**Supplementary Table2**

Gene Primer Sequences

| Genes |  | Sequences (5' - 3') |
| --- | --- | --- |
| GAPDH | Forward | GAAGGTGAAGGTCGGAGTC |
|  | Reverse | GAAGATGGTGATGGGATTTC |
| PILRA | Forward | GGCTCTCCTCACTCACCTCA |
|  | Reverse | TAGTCCGCTGCTGACCTTTC |
| LILRA2 | Forward | AGACTCCACGACTACATCCC |
|  | Reverse | CCACGCTGAAGGATGCATTG |
| MYO1F | Forward | GAGAAGGTCCAGCACGTCAA |
|  | Reverse | GGCGGGATCCCAATAACCTG |
| HPGDS | Forward | CACCAGAGCCTAGCAATAGCA |
|  | Reverse | AGTCTGCCCAAGTTACAGAGT |
| MPO | Forward | GATGTGCAACAACAGACGCA |
|  | Reverse | GAAGCCGTCCTCATACTCCG |
